# High flow nasal oxygen for acute type two respiratory failure: a systematic review

**DOI:** 10.12688/f1000research.52885.2

**Published:** 2021-09-20

**Authors:** Asem Abdulaziz Alnajada, Bronagh Blackwood, Abdulmajeed Mobrad, Adeel Akhtar, Ivan Pavlov, Murali Shyamsundar

**Affiliations:** 1 Wellcome-Wolfson Institute For Experimental Medicine, Queen's University Belfast, Belfast, UK; 2 Prince Sultan college for emergency medical services, King Saud University, Riyadh, Saudi Arabia; 3 Emergency department, Royal Victoria Hospital, Belfast, Belfast, UK; 4 Emergency department, Hôpital de Verdun, Montréal, Canada

**Keywords:** High flow nasal oxygen, high flow nasal therapy, acute type 2 respiratory failure, acute hypercapnic respiratory failure, acute exacerbation of chronic obstructive pulmonary disease

## Abstract

**Background:** Acute type two respiratory failure (AT2RF) is characterized by high carbon dioxide levels (PaCO _2 _>6kPa). Non-invasive ventilation (NIV), the current standard of care, has a high failure rate. High flow nasal therapy (HFNT) has potential additional benefits such as CO _2_ clearance, the ability to communicate and comfort. The primary aim of this systematic review is to determine whether HFNT in AT2RF improves 1) PaCO _2_, 2) clinical and patient-centred outcomes and 3) to assess potential harms.

**Methods:** We searched EMBASE, MEDLINE and CENTRAL  (January 1999-January 2021). Randomised controlled trials (RCTs) and cohort studies comparing HFNT with low flow nasal oxygen (LFO) or NIV were included. Two authors independently assessed studies for eligibility, data extraction and risk of bias. We used Cochrane risk of bias tool for RCTs and Ottawa-Newcastle scale for cohort studies.

**Results:** From 727 publications reviewed, four RCTs and one cohort study (n=425) were included. In three trials of HFNT vs NIV, comparing PaCO _2_ (kPa) at last follow-up time point, there was a significant reduction at four hours (1 RCT; HFNT median 6.7, IQR 5.6 – 7.7 vs NIV median 7.6, IQR 6.3 – 9.3) and no significant difference at  24-hours or five days. Comparing HFNT with LFO, there was no significant difference at 30-minutes. There was no difference in intubation or mortality.

**Conclusions:** This review identified a small number of studies with low to very low certainty of evidence. A reduction of PaCO _2_ at an early time point of four hours post-intervention was demonstrated in one small RCT. Significant limitations of the included studies were lack of adequately powered outcomes and clinically relevant time-points and small sample size. Accordingly, systematic review cannot recommend the use of HFNT as the initial management strategy for AT2RF and trials adequately powered to detect clinical and patient-relevant outcomes are urgently warranted.

## Introduction

### Background

Acute type two respiratory failure (AT2RF) is characterised by arterial hypercapnia (PaCO _2_ >6 kPa or >45 mmHg) and its treatment requires ventilator support in a significant proportion of cases. ^1^ Chronic obstructive pulmonary disease (COPD) is the second-most widespread disease in the UK, with 1,201,685 cases reported in 2013. Acute exacerbations of COPD (AECOPD) account for 100,000 admissions annually in England. Of these, around 20% will present with or develop hypercapnia, an indicator of increased risk of death. ^2, 3^ Development of AT2RF in patients with COPD is associated with a significantly increased risk for requiring invasive ventilation and mortality rate, ^4, 5^ with mortality rates up to 15% in patients who require admission to the intensive care unit (ICU).

The treatment of AT2RF is aimed at the underlying pathological processes such as fluid overload, bronchospasm and infection along with controlled oxygen therapy, to decrease the work of breathing. Patients often require ventilator support that may be non-invasive ventilation (NIV) or invasive mechanical ventilation (IMV). Current guidelines recommend the use of NIV. ^1^ Current evidence has established the role of NIV in improving arterial oxygenation, hypercapnia, acidosis, mortality and intubation rates. ^6^ However, the NIV failure rate ranges from 15 to 25%, with some evidence stating a failure rate as high as 60%. ^7– 10^ The factors leading to NIV failure include non-compliance due to claustrophobia, delirium, sputum retention, reduced communication and skin compromise such as skin necrosis in the nasal bridge. ^1, 10, 11^

High flow nasal oxygen or insufflation (described as high flow nasal therapy (HFNT) in this manuscript) is novel respiratory support that integrates humidified air with a high flow rate of up to 60 L/minute. Reported benefits from HFNT include consistent fractional inspired oxygen delivery, dead space washout, reduced work of breath, comfort and tolerability, ability to communicate, mucous clearance and NIV-like effects, which makes it a more tolerable method for patients. ^12– 15^ In type I ARF with different aetiologies, HFNT has been demonstrated to lead to improved oxygenation, lower rates of endotracheal intubations and lower mortality. ^16, 17^

In the last 10 years, evidence has emerged for its increasing use and a role for these modalities in clinical practice for the treatment of AT2RF. ^15, 18^ Several observational studies have suggested potential benefits of HFNT for AT2RF as demonstrated by improved gas exchange and acidosis, ^19, 20^ and reductions in the respiratory rate and work of breathing. ^21– 23^ Individual studies have shown that HFNT improves blood gas levels in AT2RF patients ^22– 25^ and is associated with improved comfort. ^24^

### Why this review is important

Adequate respiratory support through controlled oxygen, reduced work of breathing and CO _2_ clearance is essential to prevent intubation and invasive ventilation. NIV, despite its frequent use, has limitations and a high failure rate. HFNT might overcome the limitations of NIV and could be used in AT2RF patients as an initial intervention or in patients who do not tolerate NIV. Despite the increase in current literature suggesting benefits from the use of HFNT in AT2RF, current evidence is limited. Other systematic reviews are exploring the use of HFNT for the management of AT2RF post-extubation and after initial stabilization of the patient using a respiratory optimisation method like NIV or LFO. ^26- 28^ However, there is no systematic review that focuses on the use of HFNT as an initial management strategy for AT2RF.

### Objectives

The primary objective of this systematic review was to determine whether the use of HFNT for patients with AT2RF improves PaCO _2_ in comparison to LFO or NIV. Secondary objectives were to examine whether HFNT in patients with AT2RF improves other clinical or patient-centred outcomes and to assess any potential harms.

## Methods

The systematic review was registered in the PROSPERO database ( CRD42019148748, 05/09/2019) and published a priori. We conducted this systematic review according to the PRISMA guidelines (see *Reporting guidelines*). ^29, 30^

### Eligibility criteria

Randomized controlled trials, uncontrolled trials and cohort studies were included if they compared the use of HFNT with a flow rate >20 L/minutes versus LFO or NIV. We included studies of adult (≥18 years old) patients with AT2RF (>6 kPa or >45 mmHg) managed as inpatients in an acute care setting (emergency department, respiratory ward or critical care units). We excluded reports that described the use of HFNT in peri-operative settings, drug overdose, or ventilator weaning.

### Outcomes

The primary outcome for this review was the change in PaCO _2_ post-intervention (measured at time points reported by authors). The secondary outcomes were: respiratory parameters including pH, the partial arterial pressure of oxygen (PaO _2_), dyspnoea score, tidal volume and minute volume; mucous clearance (before, during or after HFNT application); the level of consciousness; patient comfort; intubation rate; length of stay in hospital; mortality; post-discharge COPD exacerbation rate and readmission rate secondary to AECOPD.

### Search strategy

We searched the electronic databases MEDLINE, EMBASE and the Cochrane Central Register of Controlled Trials from January 1999 to January 2021. The databases search was conducted on 15/01/2021. Language restrictions were not applied. In addition, we searched Google Scholar and references of all articles for any additional studies. With the assistance of a professional librarian, we developed a systematic search strategy using appropriate keywords and MeSH terms and these are detailed in the data availability section (see *Extended data*). ^30^ The systematic review software management system Covidence was used to store citations, remove duplication and aid screening.

### Selection of studies

Two review authors (AAA and MS) independently screened the titles and abstracts of all citations. The full texts of all potentially eligible studies were independently reviewed for inclusion confirmation. Any disagreement was resolved through discussion within the review team.

### Data extraction

Data were independently extracted from included studies using a standardized data extraction form by two reviewers (AAA and MS). The information extracted included type and setting of the study, recruitment information, participant characteristics (age and underlying conditions), inclusion criteria, nature of interventions, in each group (e.g. flow rate and method of delivery), time-points of measurement and outcomes. Any disagreement was resolved through discussion with BB. Data that were unavailable or insufficient from publications were requested from study authors.

Two reviewers (AAA and MS) independently assessed the quality of included studies using the Cochrane risk of bias tool for RCTs and the Newcastle-Ottawa scale for cohort studies. ^31, 32^ Each potential source of bias was marked as high, low or unclear. We assessed the quality of the evidence associated with HFNT for AT2RF using GRADE to determine the strength of the evidence into one of four grades: high, moderate, low or very low. ^33^ The quality of evidence is reported in the Summary of Findings (SOF) tables ( Tables 1, 2, 3).

**Table 1.** Summary of findings: High flow nasal therapy versus non-invasive ventilation for acute hypercapnic respiratory failure.

Patient or population: Acute hypercapnic respiratory failure patient Setting: Acute Care Intervention: HFNT Comparison: NIV					
Outcomes	Interventions		MD ^*^/ OR ^*^ (95%)/p-value	№ of participants analyzed (studies)	The certainty of the evidence (GRADE)
	NIV ^*^ Median (IQR ^*^) or mean (SD ^*^)	HFNT ^*^ Median (IQR ^*^) or mean (SD ^*^)			
Primary outcome a. PaCO _2_ (kPa ^*^) ^34^ time-point: 4 hours	7.6 (6.3 – 9.3)	6.7 (5.6 – 7.7)	P = 0.03	65 (1 RCT)	⊕⊕⊕O Moderate _2_
b. PaCO _2_ (kPa ^*^) ^36^ time-point: 6 hours	7.7 (1.6)	8.5 (2)	MD 0.80 [0.00, 1.60]	88 (1 RCT)	⊕OOO Very Low _2,4_
c. PaCO _2_ (kPa ^*^) ^23^ time-point: 24 hours	6.6 (1.9)	6.3 (2.1)	MD −0.30 [−1.14, 0.54]	88 (1 Cohort)	⊕OOO Very Low _2,3_
d. PaCO _2_ (kPa ^*^) ^35^ time-point: 5 days	8 (1.9)	7.8 (1.9)	MD −0.20 [−0.77, 0.37]	165 (1 RCT)	⊕⊕⊕O Moderate _2_
Secondary outcome (continuous data) a. PaO _2_ (kPa ^*^) ^34^ time-point: 4 hours	11.7 (10.3 – 12.9)	11.1 (5.3 – 13.2)	P = 0.71	65 (1 RCT)	⊕⊕⊕O Moderate _2_
b. PaO _2_ (kPa ^*^) ^36^ time-point: 6 hours	^‡^N/R	^‡^N/R	^‡^N/R	88 (1 RCT)	⊕OOO Very Low _2,4_
c. PaO _2_ (kPa ^*^) ^23^ time-point: 24 hours	11.3 (3.1)	11.2 (2.5)	MD −0.10 [−1.28, 1.08]	88 (1 Cohort)	⊕OOO Very Low _2,3_
d. PaO _2_ (kPa ^*^) ^35^ time-point: 5 days	^‡^11 (2.1)	^‡^10.9 (2)	^‡^MD −0.10 [0.72, 0.52]	165 (1 RCT)	⊕⊕⊕O Moderate _2_
e. pH ^34^ time-point: 4 hours	7.35 (7.3-7.4)	7.4 (7.3-7.4)	P = 0.24	65 (1 RCT)	⊕⊕⊕O Moderate _2_
f. pH ^36^ time-point: 6 hours	N/R ^‡^	N/R ^‡^	N/R ^‡^	88 (1 RCT)	⊕OOO Very Low _2,4_
g. pH ^23^ time-point: 24 hours	7.4 (0.1)	7.4 (0.1)	MD 0.00 [−0.03, 0.03]	88 (1 Cohort)	⊕⊕OO Low _3_
h. pH ^35^ time-point: 5 days	7.4 (0.1) ^‡^	7.35 (0.1) ^‡^	MD −0.05 [0.08, 0.01] ^‡^	165 (1 RCT)	⊕⊕⊕O Moderate _2_
GRADE Working Group grades of evidence High certainty: We are very confident that the true effect lies close to that of the estimate of the effect Moderate certainty: We are moderately confident in the effect estimate: The true effect is likely to be close to the estimate of the effect, but there is a possibility that it is substantially different Low certainty: Our confidence in the effect estimate is limited: The true effect may be substantially different from the estimate of the effect Very low certainty: We have very little confidence in the effect estimate: The true effect is likely to be substantially different from the estimate of effect					
Explanation 1- Downgraded for indirectness because of the study population 2- Downgraded for imprecision for a wide confidence interval, the small sample size 3- Downgraded for study design (non-RCT) 4- Downgraded for risk of bias					

^^*^^ Abbreviations: HFNT, High flow nasal therapy; IQR, Interquartile range; kPa, Kilopascal; MD, mean difference; NIV, Non-invasive ventilation; OR, Odd ratio; SD, Standard deviation.

^^‡^^ N/R - Not reported.

**Table 2.** Summary of findings: High flow nasal therapy versus non-invasive ventilation for acute hypercapnic respiratory failure.

Patient or population: Acute hypercapnic respiratory failure patient Setting: Acute Care Intervention: HFNT Comparison: NIV						
Outcomes	Interventions		MD ^*^/OR ^*^ (95%)/p-value	№ of participants analyzed (studies)	The certainty of the evidence (GRADE)	Comments
	NIV ^*^ n/N ^*^	HFNT ^*^ n/N ^*^				
Secondary outcome (Dichotomous data) a. Mortality rate ^23^ time-point: 30-day	44/8	44/7	OR 0.85 [0.28, 2.59]	88 (1 Cohort)	⊕⊕OO Low _3_	
b. Mortality rate ^36^ time-point: in-hospital mortality	39/6	40/2	OR 0.29 [0.05, 1.53]	80 (1RCT)	⊕⊕⊕O Moderate _2_	The study didn’t provide the time-point for mortality.
c. Intubation rate ^36^ time-points: 6 hours	39/1	40/1	OR 0.97 [0.06, 16.14]	80 (1RCT)	⊕⊕⊕O Moderate _2_	
d. Intubation rate ^34^ time-point: 72 hours	31/5	34/2	OR 0.33 [0.06, 1.81]	65 (1 RCT)	⊕⊕⊕O Moderate _2_	
e. Intubation rate ^23^ time-points: 30-day	44/12	44/11	OR 0.89 [0.34, 2.30]	88 (1 Cohort)	⊕⊕OO Low _3_	
f. Dysponea ^34, 36^	-	-	-	145 (2RCTs)	-	Dyspnoea was measured by 2 studies at various time points. It was assessed by modified Borg.
g. Patient comfort ^35, 36^	-	-	-	245 (2 RCTs)	-	Comfort was measured by 2 studies at various time points. It was assessed by a self-designed survey and a 10-point numerical rating scale.
GRADE Working Group grades of evidence High certainty: We are very confident that the true effect lies close to that of the estimate of the effect Moderate certainty: We are moderately confident in the effect estimate: The true effect is likely to be close to the estimate of the effect, but there is a possibility that it is substantially different Low certainty: Our confidence in the effect estimate is limited: The true effect may be substantially different from the estimate of the effect Very low certainty: We have very little confidence in the effect estimate: The true effect is likely to be substantially different from the estimate of effect						
Explanation 1- Downgraded for indirectness because of the study population 2- Downgraded for imprecision for a wide confidence interval, the small sample size 3- Downgraded for study design (non-RCT) 4- Downgraded for risk of bias						

^*^ Abbreviations: HFNT, High flow nasal therapy; IQR, Interquartile range; MD, mean difference; n/N, Number of patients NIV, Non-invasive ventilation; OR, Odd ratio; SD, Standard deviation.

**Table 3.** Summary of findings: High flow versus low flow nasal therapy for acute hypercapnic respiratory failure.

Patient or population: Acute hypercapnic respiratory failure patient Setting: Acute Care Intervention: HFNT Comparison: LFO						
Outcomes	Interventions		MD ^*^ (95%)	№ of participants analysed (studies)	The certainty of the evidence (GRADE)	Comments
	LFO ^*^ Mean (SD ^*^)	HFNT ^*^ Mean (SD ^*^)				
Primary outcome a. PaCO _2_ (KPa ^*^) ^24^ time-point: 30 minutes	6.5 (1.3)	6.3 (1.3)	-0.20 [-1.24, 0.84]	24 (1 RCT)	⊕OOO Very low _2,4_	
Secondary outcome (continuous) a. Patient comfort ^24^	-	-	-	24 (1 RCT)	⊕OOO Very Low _2,4_	The questions used to assess patient comfort were not validated, and the sample size was low
GRADE Working Group grades of evidence High certainty: We are very confident that the true effect lies close to that of the estimate of the effect Moderate certainty: We are moderately confident in the effect estimate: The true effect is likely to be close to the estimate of the effect, but there is a possibility that it is substantially different Low certainty: Our confidence in the effect estimate is limited: The true effect may be substantially different from the estimate of the effect Very low certainty: We have very little confidence in the effect estimate: The true effect is likely to be substantially different from the estimate of effect						
Explanation 1- Downgraded for serious indirectness because of the study population 2- Downgraded for serious imprecision for a wide confidence interval, the small sample size 3- Downgraded for study design (non-RCT) 4- Downgraded for risk of bias						

^*^ Abbreviations: HFNT, High flow nasal therapy; LFO, kPa, Kilopascal; Low flow oxygen; MD, mean difference; SD, Standard deviation.

### Data synthesis

*Measurement of effect*

RevMan software (Review Manager, version 5.3) was used for data analysis. Results are reported as odds ratios (ORs) with 95% confidence intervals (CIs) for binary variables and mean differences (MD) with 95% CIs for continuous variables. A meta-analysis was planned, but there were insufficient studies and results are presented narratively.

*Subgroup and sensitivity analysis*

The planned subgroup analyses of patient conditions (COPD, neuromuscular disorders, and interstitial lung disease), and the planned sensitivity analysis excluding trials with a high risk of bias could not be undertaken due to the low number of trials.

## Results

**Figure 1.**
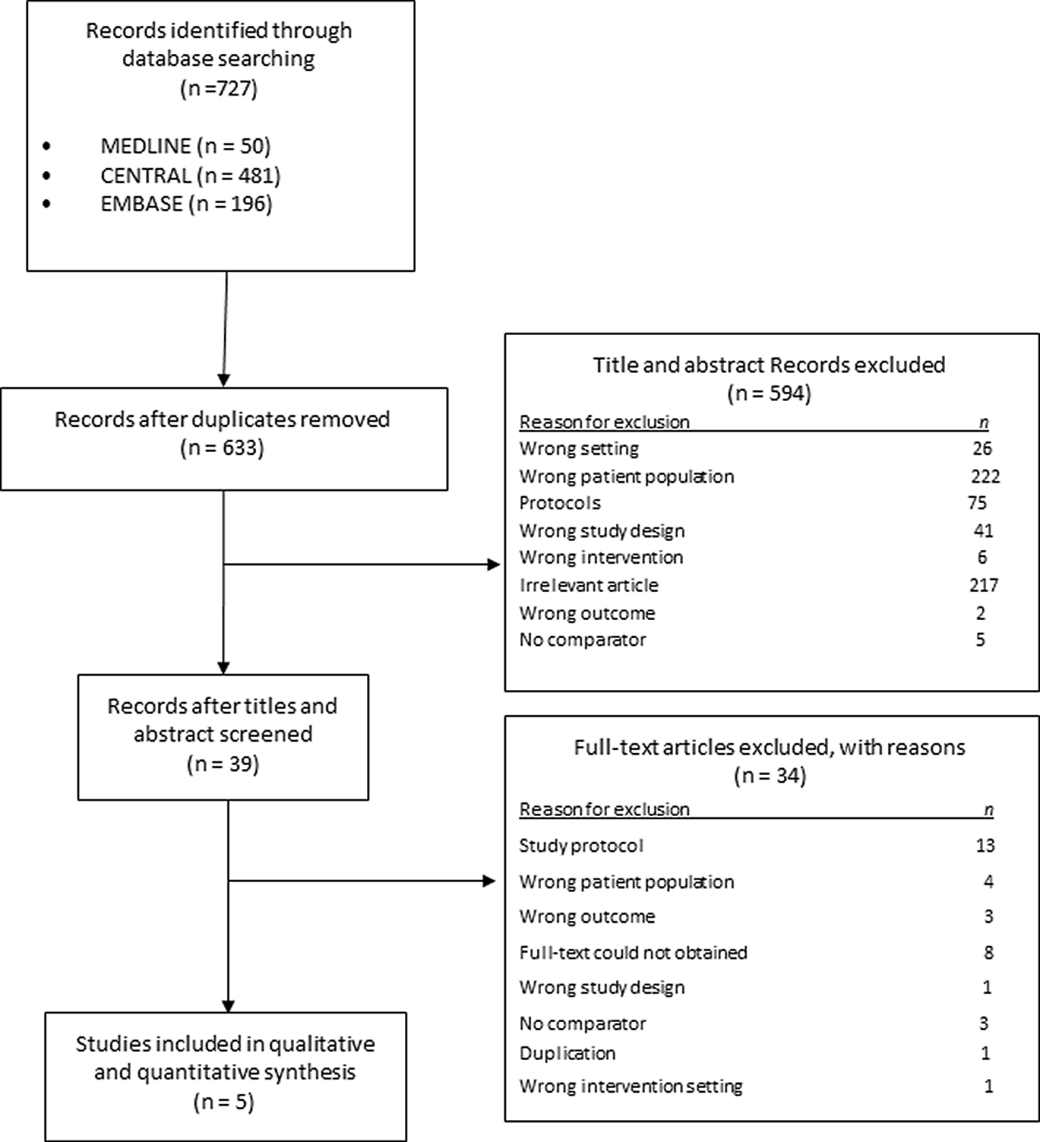
PRISMA flowchart for study selection.

The search identified 727 records. Following the removal of duplicates and non-eligible studies, 39 full-text studies were screened and 34 studies were excluded. Five studies with 425 participants were included in this review ( Figure 1). ^23, 24, 34– 36^

### Study characteristics and risk of bias

The characteristics of the included studies are summarized in Table 4. Four studies were RCTs ^23, 24, 34– 36^ and one was a cohort study. ^23^ Four studies compared HFNT with NIV ^23, 34– 36^ and one RCT compared HFNT with LFO using simple nasal prongs. ^24^ The disease state of interest was an acute-moderate hypercapnic respiratory failure (n = 88) in one study, ^23^ and AECOPD (n = 337) in the remaining four studies. ^24, 34– 36^

**Table 4.** Study characteristics.

Author and year	No. of participants	Country	Setting	Study design	Population	Intervention (flow rate L/min)	Control	Outcomes measured relevant for this review
Cortegiani *et al*. ^36^ 2020	80	Italy	Emergency Department, Intensive Care Units or Respiratory Unit	RCT ^*^	AECOPD ^*^	HFNT ^*^ (60 L/min)	NIV ^*^	PaCO _2_, PaO _2_, pH, intubation rate, mortality, dyspnoea score, comfort, hospital stay
Doshi *et al*. ^34^ 2020	65	United States of America	Emergency department	RCT ^*^	AECOPD ^*^	HFNT ^*^ (35 L/min)	NIV ^*^	PaCO _2_, PaO _2_, pH, dyspnoea score, intubation rate, hospital stay
Cong *et al*. ^35^ 2019	168	China	Intensive care unit	RCT ^*^	AECOPD ^*^	HFNT ^*^ (30 – 35 L/min)	NIV ^*^	PaCO _2_, PaO _2_, pH, comfort, hospital stay
Lee *et al*. ^23^ 2018	88	South Korea	Emergency department	Cohort	Acute-moderate hypercapnic respiratory failure	HFNT ^*^ (35 L/min)	NIV ^*^	PaCO _2_, PaO _2_, pH, intubation rate, mortality
Pilcher *et al*. ^24^ 2017	24	New Zealand	Emergency department	RCT ^*^	AECOPD ^*^	HFNT ^*^ (35 L/min)	Standard nasal prong	PaCO _2_, patient tolerability

^*^ Abbreviations: ACOPD, Acute chronic obstructive pulmonary disease; COPD, Chronic obstructive pulmonary disease; HFNT, High flow nasal therapy; n/N: Number of patients; NIV: Non-invasive ventilation; RCT, Randomized controlled trial.

**Figure 2.**
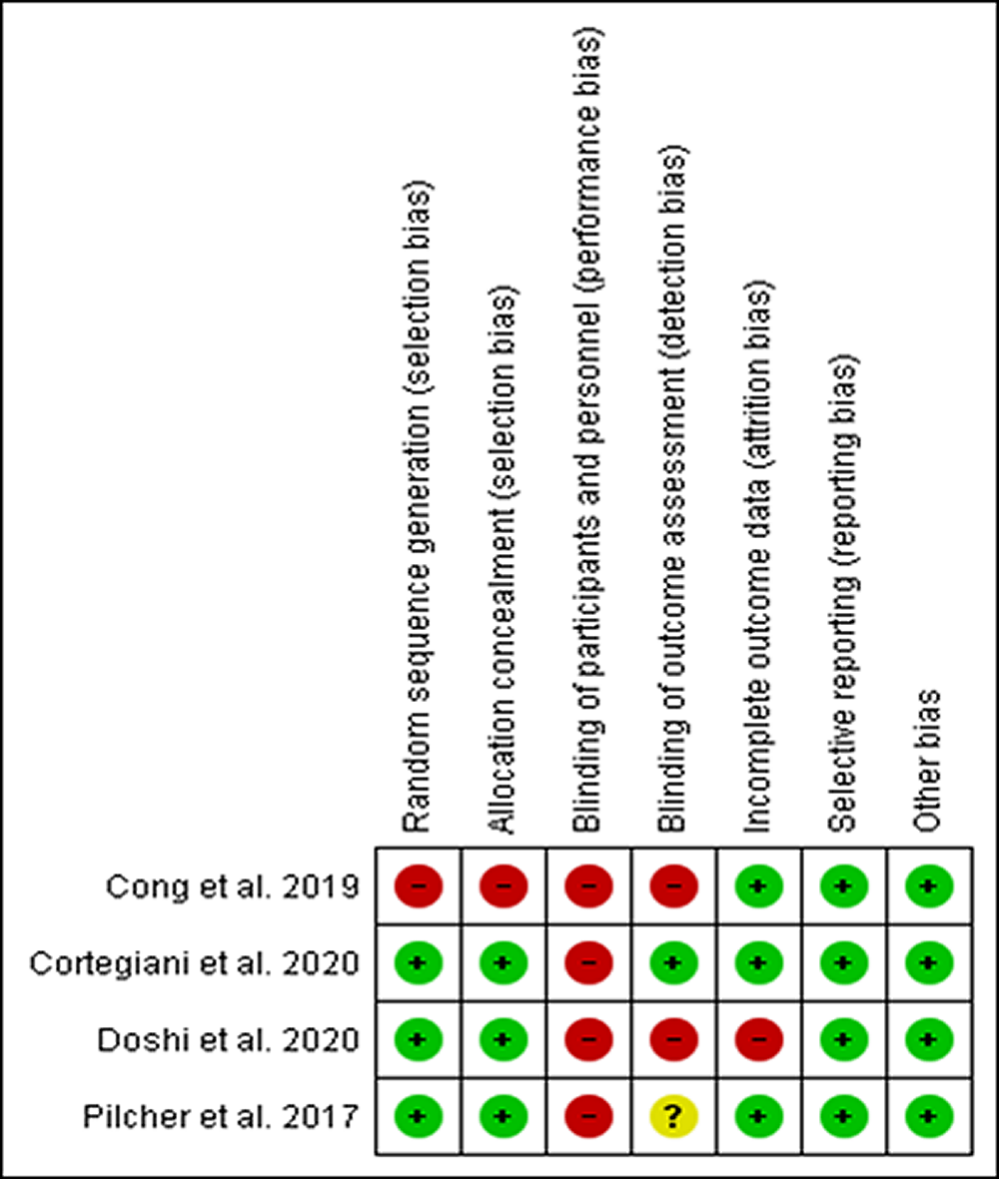
Risk of bias assessment.

The risk of bias assessments for the four RCTs are described in Figure 2. ^24, 34– 36^ Blinding of participants and personnel were not possible in the trials. One trial showed a high risk for selection bias due to unexplained randomization sequence and allocation concealment. ^35^ The trials showed a high risk or unclear risk of detection bias due to no ^34, 35^ or unclear ^24^ blinding of the outcomes assessor. One trial showed a high risk of attrition bias due to unreported incomplete data. ^34^ The cohort study showed a low risk of bias in all domains and did not describe how the outcomes were assessed. ^23^

### Primary outcome (PaCO _2_)

**Figure 3.**
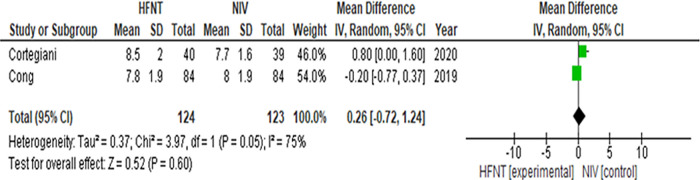
Forest plot of PaCO _2_ at last available time-points from Cong *et al*. ^35^ and Cortegiani *et al*. ^36^

Changes in PaCO _2_ after the intervention was reported in all five studies ( Table 5), ^23, 24, 34– 36^ four studies compared HFNT to NIV. ^23, 34– 36^ Doshi *et al*. ^34^ reported no significant difference in PaCO _2_ at one hour between HFNT and NIV but there was a significant reduction in PaCO _2_ at four hours (HFNT 6.7, 5.6 – 7.7 vs NIV 7.6, 6.3 – 9.3 (Median, interquartile range (IQR)). In the other studies comparing HFNT to NIV, ^23, 35, 36^ there was no significant difference in PaCO _2_ at various time-points with a similar trend in PaCO _2_ ( Figure 3). Pilcher *et al*. ^24^ compared HFNT with LFO at various five-minute time intervals with no significant difference, but when adjusted for the baseline PaCO _2_, they reported a significant improvement in PaCO _2_ by HFNT when compared to LFO.

**Table 5.** Trends in PaCO2 (kPa) at various time-points.

Study	Time-points	HFNT ^*^ n/N ^*^	HFNT ^*^ Mean (SD ^*^) or Median (IQR ^*^)	n/N ^*^		Mean (SD ^*^) or Median (IQR ^*^)		Mean difference
				NIV ^*^	LFO ^*^	NIV ^*^	LFO ^*^	
Doshi *et al*. ^34^ 2020	Baseline	34/34	56 (26 – 112)	34/31	-	64.6 (38 – 137)	-	-
	1 hour	34/33	56 (23 – 130)	34/31	-	63 (31 – 122)	-	-
	4 hour	34/27	50 (31 – 74)	34/25	-	57 (35 – 113)		
Cortegiani *et al*. ^36^ 2020	Baseline	80/40	9.8 (1.7)	80/39	-	9.6 (1.7)	-	0.20 [−0.55, 0.95]
	2 hours	80/40	9.1 (2.1)	80/39	-	8.4 (1.8)	-	0.70 [−0.16, 1.56]
	6 hours	80/40	8.5 (2)	80/39	-	7.7 (1.6)	-	0.80 [0.00, 1.60]
Cong *et al*. ^35^ 2019	Baseline	168/84	9.6 (2.2)	168/84	-	9.6 (2.3)	-	0.00 [−0.68, 0.68]
	12 hours	168/84	8.4 (2.1)	168/84	-	8.4 (2.1)	-	0.00 [−0.64, 0.64]
	5 days	168/84	7.8 (1.9)	168/84	-	8 (1.9)	-	−0.20 [−0.77, 0.37]
Lee *et al*. ^23^ 2018	Baseline	88/44	7.50 (1.3)	88/44	-	7 (1.2)	-	0.50 [−0.02, 1.02]
	6 hours	88/44	6.20 (2.2)	88/44	-	6.90 (2.3)	-	−0.70 [−1.64, 0.24]
	24 hours	88/44	6.30 (2.1)	88/44	-	6.6 (1.9)	-	−0.30 [−1.14, 0.54]
Pilcher *et al*. ^24^ 2017	Baseline	24/12	6.50 (1.3)	-	24/12	-	6.50 (1.3)	0.00 [−1.04, 1.04]
	5 minutes	24/12	6.40 (1.3)	-	24/12	-	6.50 (1.3)	−0.10 [−1.14, 0.94]
	10 minutes	24/12	6.30 (1.3)	-	24/12	-	6.50 (1.3)	−0.20 [−1.24, 0.84]
	15 minutes	24/12	6.30 (1.3)	-	24/12	-	6.50 (1.3)	−0.20 [−1.24, 0.84]
	20 minutes	24/12	6.35 (1.3)	-	24/12	-	6.40 (1.3)	−0.05 [−1.09, 0.99]
	25 minutes	24/12	6.40 (1.3)	-	24/12	-	6.40 (1.3)	0.00 [−1.04, 1.04]
	30 minutes	24/12	6.30 (1.3)	-	24/12	-	6.50 (1.3)	−0.20 [−1.24, 0.84]

^*^ Abbreviations: RCT, Randomized controlled trial; HFNT, High flow nasal therapy; NIV, Non-invasive ventilation; LFO, Low flow oxygen; n/N, Number of patients; SD, Standard deviation; min, minutes.

### Secondary outcomes

pH level was reported in three studies. ^23, 34, 35^ Doshi *et al*. ^34^ reported no significant difference in pH between HFNT and NIV at one hour (HFNT 7.36, 7.34-7.42 vs NIV 7.31, 7.27-7.37, (Median, IQR)) or four hours (HFNT 7.38, 7.34-7.42 vs NIV 7.35, 7.33-7.37, (Median, IQR)). Cong *et al*. ^35^ reported no significant difference in pH between HFNT and NIV at 12 hours (MD -0.10, 95% CI -0.13, 0.06) or five days (MD -0.05, 95% CI -0.08, -0.01). Lee *et al*. ^23^ showed no significant difference in pH between HFNT and NIV at six hours (MD 0.02, 95% CI -0.16, 0.20) or 24 hours (MD 0.00, 95% CI -0.03, 0.03). The PaO _2_ level was reported in four trials. ^23, 34– 36^ Doshi *et al*. ^34^ reported no significant difference in PaO _2_ between HFNT and NIV at one hour (HFNT 13.2, 10.7-19.2 vs NIV 15.1, 8.8-22.9, (Median, IQR)) or four hours (HFNT 11.1, 5.3-13.2 vs NIV 11.7, 10.3-12.9, (Median, IQR)). Cong *et al*. ^35^ reported no significant difference in PaO _2_ between HFNT and NIV at 12 hours (MD 0.00 kPa, 95% CI −0.70, 0.70) or five days (MD −0.10 kPa, 95% CI −0.72, 0.52). Lee *et al*. ^23^ reported no significant difference in PaO _2_ between HFNT and NIV at six hours (MD 0.00 kPa, 95% CI −1.30, 1.30) or 24 hours (MD −0.10 kPa, 95% CI −1.28, 1.08).

Patient comfort was reported in three RCTs. ^24, 35, 36^ Patient comfort assessed using a self-designed survey in Cong *et al*. ^35^ a 10-point numerical rating scale in Cortegiani *et al*. ^36^ and the Likert scale in Pilcher *et al*. ^24^ showed that HFNT was more comfortable than LFO but louder than LFO ( Table 6).

**Table 6.** Comfort score using Likert scale ^*^ (RCT comparing HFNT vs SNP). ^24^

Study	Time-points	Question	HFNT ^‡^ n/N ^‡^	Mean (SD ^‡^) HFNT ^‡^	SNP ^‡^ N/n ^‡^	Mean (SD ^‡^) SNP ^‡^	MD ^‡^
Pilcher *et al*. ^24^ 2017	30 minutes	I found wearing the nasal interface: *1 = Very comfortable* *5 = Very uncomfortable*	24/12	2.4 (1.3)	24/12	2.4 (1.1)	0.00 [−0.96, 0.96]
		The nasal interface was: *1 = Light* *5 = Heavy*	24/12	2.2 (1.2)	24/12	1.9 (1.2)	0.30 [−0.66, 1.26]
		The intervention was: *1 = Quiet* *5 = Noisy*	24/12	2.6 (1.4)	24/12	1.3 (0.6)	1.30 [0.44, 2.16]
		My nasal passages were: *1 = Comfortable* *5 = Dry*	24/12	1.9 (1.2)	24/12	3.0 (1.7)	−1.10 [−2.28, 0.08]
		Breathing through my nose was: *1 = Easy* *5 = Very difficult*	24/12	2.3 (1.2)	24/12	1.8 (1.0)	0.50 [−0.38, 1.38]

^*^ Answers to questions were made on a 1-5.

^‡^ Abbreviations: RCT, Randomized controlled trial; HFNT, High flow nasal therapy; n/N, Number of patients; SD, Standard deviation; NIV, Non-invasive ventilation; MD, mean difference; SNP, Simple nasal prongs; MD, mean difference.

The intubation rate was reported in three studies comparing HFNT with NIV. ^23, 34, 36^ Doshi *et al*. ^34^ demonstrated no significant difference in intubation rate at 72 hours (RCT; OR 0.33 95% CI 0.06, 1.81). Cortegiani *et al*. ^36^ reported no significant difference in intubation rate at two hours (RCT; OR 0.32 95% CI 0.01, 8.02) or six hours (RCT; OR 0.97 95% CI 0.06, 16.14). Lee *et al*. ^23^ reported no significant difference at 30 days (cohort; OR 0.89 95% CI 0.34, 2.30) ( Table 7).

**Table 7.** Comfort score using 10-point numerical rating scale ^*^ (RCT comparing HFNT vs NIV). ^36^

Study	Time-points	HFNT ^‡^			NIV ^‡^			P-value
		n/N ^‡^	Median	IQR ^‡^	n/N ^‡^	Median	IQR ^‡^	
Cortegiani *et al*. ^36^ 2020	2 hours	80/40	1	[0–2]	80/39	3	[1–5]	0.0010
	6 hours	80/40	0	[0–2]	80/39	2	[1–4]	0.0003

^*^ 10-point numerical rating scale: where 0 is no discomfort and 10 is maximum discomfort

^‡^ Abbreviations: HFNT, High flow nasal therapy; n/N, Number of patients; NIV, Non-invasive ventilation; IQR, Interquartile range.

The mortality rate was reported in two studies ^23, 36^ and there was no difference between HFNT and NIV groups ( Table 7).

The dyspnoea score, measured by Modified Borg score, a self-reported rating of perceived dyspnoea on a scale of one to 10, with 10 being the worst, was reported in two trials. ^34, 36^ The reduction in the dyspnoea score was similar between HFNT and NIV at different time points in both trials ( Table 8).

**Table 8.** Comfort score using self-designed survey* (comparing HFNT vs NIV). ^35^

Study	Time-points	Treatment	n/N ^‡^	Comfort N (%)
Cong *et al*. ^35^ 2019	12 hours and 5 days	NIV ^‡^	168/84	57 (67.9)
		HFNT ^‡^	168/84	75 (88.2)
		P-value	0.008	

Self-designed survey: developed by the researchers to measure the comfort and satisfaction of patients in both groups.

^‡^ Abbreviations: High flow nasal therapy; n/N, Number of patients; NIV, Non-invasive ventilation

Length of stay in hospital was reported by three trials ^34– 36^ comparing HFNT and NIV with no difference between the two groups ( Table 9).

**Table 9.** Mortality and intubation rate.

Study	Outcome	Time-points	HFNT ^*^ n/N ^*^	NIV ^*^ n/N ^*^	OR ^*^ (95%)
Lee *et al*. ^23^ 2018	Mortality rate	30-day	44/7	44/8	0.85 [0.28, 2.59]
Cortegiani *et al*. ^36^ 2020	Mortality rate	In hospital mortality	40/2	39/6	0.29 [0.05, 1.53]
Doshi *et al*. ^34^ 2020	Intubation rate	72-hours	34/2	31/5	0.33 [0.06, 1.81]
Cortegiani *et al*. ^36^ 2020	Intubation rate	2 hours	40/0	39/1	0.32 [0.01, 8.02]
		6 hours	40/1	39/1	0.97 [0.06, 16.14]
Lee *et al*. ^23^ 2018	Intubation rate	30-days	44/11	44/12	0.89 [0.34, 2.30]

^*^ Abbreviations: HFNT, High flow nasal therapy; n/N: Number of patients; NIV: Non-invasive ventilation; OR: Odd ratio

**Table 10.** Dyspnoea score using Modified Borg Score ^*^ (comparing HFNT vs NIV). ^33, 36^

Study	Time points	HFNT ^‡^			NIV ^‡^	P-value/MD ^‡^
		n/N ^‡^	Median (IQR ^‡^) /Mean (SD ^‡^)	n/N ^‡^	Median (IQR ^‡^)/Mean (SD ^‡^)	
Doshi *et al*. ^34^ 2020	30 minute	65/33	4 (3-7)	65/29	4 (2-6)	451
	1 hour	65/31	3 (2-6)	65/29	3 (1.5-5)	0.595
	90 minute	65/31	3 (2-5)	65/29	2 (0-4.5)	0.11
	4 hours	65/28	2 (1-3.75)	65/24	3 (1-4)	0.788
Cortegiani *et al*. ^36^ 2020	2 hours	80/40	3 (2)	80/39	3 (2)	0.00 [−0.88, 0.88]
	6 hours	80/40	5 (2)	80/39	5 (2)	0.00 [−0.88, 0.88]

^*^ Borg Modified Score: a self-reported rating of perceived dyspnoea on a scale of one to 10, with 10 being the worst.

^‡^ Abbreviations: n/N, Number of patients; IQR, Interquartile range; HFNT, High flow nasal therapy; NIV, Non-invasive ventilation

**Table 11.** Length of stay.

Study	HFNT ^*^ n/N ^*^	Mean SD ^*^/Median IQR ^*^	NIV ^*^ n/N ^*^	Mean (SD ^*^)/Median (IQR ^*^)	MD ^*^
Doshi *et al*. ^34^ 2020	65/34	105.1 hours (78.5-178.3)	65/31	120.4 hours (67-144.5)	-
Cortegiani *et al*. ^36^ 2020	80/40	10 days (9-19)	80/39	13 days (9-16)	-
Cong *et al*. ^35^ 2019	168/84	18.04 (6.15)	168/84	18.31 (7.01)	−0.27 days

^*^ Abbreviations: n/N, Number of patients; IQR, Interquartile range; HFNT, High flow nasal therapy; NIV, Non-invasive ventilation; MD, mean difference; SD, standard deviation

## Discussion

Within the AT2RF patient population where HFNT is used as the initial management strategy, this systematic review has identified very few studies: four comparing HFNT with NIV and one comparing HFNT with LFO. HFNT, compared with NIV, showed a significant difference in PaCO _2_ after four hours of treatment, ^34^ although the difference was not demonstrated at 24 hours, ^23^ five days, ^35^ six hours ^36^ and a similar lack of difference is seen when compared to LFO at 30 minutes. ^24^ The reduction in PaCO2 between the two groups at four hours demonstrated in Doshi *et al*. ^34^ is not adjusted for the baseline difference in PaCO _2_ between the two groups. The absolute reduction of PaCO _2_, when compared to the baseline, was 0.8 kPa for the HFNT group and 0.99 kPa in the NIV group, which suggest that the significant difference was secondary to baseline difference rather than true clinical superiority. Compared with NIV or LFO, HFNT showed no difference in pH and PaO _2_ and has similar intubation rates, mortality and hospital length of stay. HFNT, when compared to NIV, is associated with better comfort as presented by Cong *et al*. ^35^ and Cortegiani *et al*., ^36^ although this was not replicated in Pilcher *et al*. ^24^ This systematic review found that despite the potential benefit of improved patient comfort and increasing use of HFNT in the treatment of AT2RF, the current evidence is quite poor. The certainty of the evidence was primarily impacted by the small number of trials and sample sizes, selection bias and few RCTs. Lack of blinding is a potential source of bias but the nature of the intervention precludes blinding, while the objective nature of the outcome measures reduces the risk of bias. Hence, objective outcome measures were not downgraded for lack of blinding while subjective measures such as comfort score and dyspnoea score were downgraded.

In AT2RF, the production of CO _2_ is increased due to additional work of breathing, increased metabolism and failure to clear CO _2_. NIV failure occurs in a quarter of these patients needing further IMV. The extent of reduction in pH, associated with the elevated CO _2_, is significantly associated with NIV failure. ^37^ Any medical optimization introduced early after the detection of AT2RF should be aimed at improving CO _2_ clearance and pH because the development of respiratory acidaemia post-admission is associated with a mortality of 33%. ^3^ While current evidence has convincingly established the benefits of NIV for AT2RF, evidence for newer and better-tolerated technologies to reduce hypercapnia is urgently required due to the high intolerance rate leading to a late failure. ^10^

In this systematic review focused on early intervention for AT2RF patients, there is no difference in various respiratory parameters between HFNT and NIV except for one study showing an improvement in PaCO _2_ at a single time-point. HFNT is associated with a reduction in PaCO _2_ and an increase in pH similar to NIV. While this could suggest that HFNT is non-inferior to NIV, HFNT cannot be recommended as an alternative management strategy to reduce PaCO _2_ due to the low quality of evidence, lack of standardization of time-points for PaCO _2_ measurement and the lack of adequately powered sample sizes. Similarly, in patients failing NIV due to compliance issues, HFNT may be a promising option to limit mechanical ventilation. This recommendation falls beyond the scope of this systematic review and is a clinical scenario that requires urgent attention. A similar response in CO _2_ to HFNT is reported in COPD patients with stable type 2 respiratory failure, ^38^ post-acute NIV, ^39^ post NIV failure, ^40– 42^ post-extubation ^43^ and during breaks in NIV. ^21^

Studies have shown a reduction in intubation rate and mortality between NIV versus usual care ^39^ and a reduction in the length of hospital stay, lower incidence of complications with a longer-term benefit of fewer readmissions to hospital in the following year between NIV and IMV ^44^ with one study suggesting a mortality benefit. ^45^ HFNT, if equivalent to NIV, should ultimately reduce important outcomes such as intubation, mortality and health resource use. Three studies found no difference in intubation rate ^23, 34, 36^ and three studies found no difference in length of stay ^34– 36^ thus suggesting therapeutic equivalence but the studies were not powered for these outcomes. Doshi *et al*., ^34^ showed that HFNT when compared to NIV had a similar therapy failure rate of approximately 25%. Patients receiving HFNT had a trend towards a shorter ICU stay, likely driven by a lower intubation rate in the HFNT group (5.9%) when compared to the NIV group (16.1%), which did not achieve statistical significance in this study that was not powered for this outcome.

A key balancing outcome is an increase in adverse outcomes that have been highlighted in studies comparing NIV to usual care that include a delay in escalation to IMV, ^46^ increased mortality when compared to immediate IMV, and increased mortality when IMV is delayed. ^47^ In this systematic review, Lee *et al*. ^23^ and Cortegiani *et al*. ^36^ taken together with lower intubation rate in the HFNT arm, ^34^ suggests that HFNT is unlikely to be associated with harm through delayed initiation of IMV, but this hypothesis needs to be confirmed in a clinical trial.

One of the putative benefits of HFNT is patient comfort due to the lack of a tight-fitting mask, prevention of skin breakdown, better communication and mucous clearance. ^24^ HFNT, when compared to NIV, was shown to be associated with improved comfort in Cong *et al.* ^35^ and Cortegiani *et al*. ^36^ In this review, Doshi *et al.* ^34^ and Cortegiani *et al*. ^36^ did not detect any difference in dyspnoea between HFNT and NIV. The lack of demonstrable benefit is likely secondary to the earlier time points in the studies investigating the role of HFNT in the initial management of AT2RF.

HFNT is increasingly emerging as a therapeutic option for AT2RF, but various studies have combined it with other clinical scenarios such as post-extubation, ^42^ NIV interruption, ^21^ or physiological studies ^24^ and even in studies that explored its efficacy in acute exacerbations, the place of intervention could lead to bias, for example after initial management in the emergency medicine department, thus introducing unintentional bias such as lead-time bias as well selection bias. ^35^ The location of patients in a closely monitored environment, as opposed to a general ward, ^47^ might mask any adverse outcomes due to deterioration through earlier intervention. Hence, it is essential to investigate its utility in the early management of AT2RF in the emergency medicine department.

High flow nasal cannula can flush anatomical dead space, provide mild positive distending pressure, improve mucociliary clearance as well as be better tolerated. ^48^ Depending on the type of respiratory failure, type 1 or 2, a specific nasal cannula design that alters flow pattern could have a differential effect. A small-bore nasal cannula as seen in high flow nasal insufflation might purge the anatomical dead space more efficiently, thereby providing minimal ventilator assistance. ^48, 49^

The strength of the systematic review is that it was conducted to a high standard following recommended methods for the conduct, quality assessment and reporting, ^50^ using a comprehensive search strategy of all electronic databases. Despite this, the recommendations of the review are limited by the small number of trials, which highlights the need for further adequately powered trials.

We recommend that future research needs to address the following research gaps in the evidence base for the use of HFNT in AT2RF. Future trial designs should be randomized controlled trials, they should include sufficiently large patient numbers to ensure they are adequately powered for important clinical outcomes. Outcomes should be standardized with clear definitions including clinical outcomes, use validated scales and relevant time points. ^51^ The role of nasal cannula diameter in the efficiency of CO _2_ clearance should be tested to determine whether the type of device used has an impact on therapy efficacy. ^52^ Studies should also encompass a robust health economic analysis, include outcome analysis of patients who fail therapy and identify any features to predict the outcome of the therapy to allow patient selection.

In conclusion, this review found very few studies investigating the clinical efficacy of HFNT in AT2RF. A similar reduction in PaCO _2_ was seen between HFNT and NIV at various time-points, ^35, 36^ while a significantly higher PaCO _2_ clearance with HFNT, when compared to NIV, was demonstrated at an early time point in one study. ^34^ Similarly, HFNT use was associated with better comfort in two studies, ^35, 36^ while a similar benefit was not shown in the other study. ^24^ The evidence is also moderate in quality and the benefit demonstrated is limited to clinically irrelevant time points, with no studies powered to detect clinical outcomes. Therefore, a change in practice cannot be recommended until further high-quality clinical trials are conducted.

## Data availability

### Underlying data

All data underlying the results are available as part of the article and no additional source data are required.

### Extended data

Queen’s University Belfast institutional data repository (Pure system): High flow nasal therapy for acute type 2 respiratory failure: A systematic review. https://doi.org/10.17034/4080c4eb-38f0-4c02-91ee-37129ceb65a6. ^30^

This project contains the following extended data:

- Search strategy for Medline for research article High flow nasal therapy for acute type two respiratory failure. A systematic review.pdf

Data are available under the terms of the Creative Commons Attribution 4.0 International license (CC-BY 4.0).

## Reporting guidelines

Queen’s University Belfast institutional data repository (Pure system): PRISMA checklist for “High flow nasal therapy for acute type 2 respiratory failure: A systematic review”. https://doi.org/10.17034/4080c4eb-38f0-4c02-91ee-37129ceb65a6. ^30^

Data are available under the terms of the Creative Commons Attribution 4.0 International license (CC-BY 4.0).
