# Supplementary material for: High flow nasal oxygen for acute type two respiratory failure: a systematic review
Source: F1000Res. 2021 Sep 20;10:482. Originally published 2021 Jun 16. [Version 2] doi: 10.12688/f1000research.52885.2 (PMC8453312; doi:10.12688/f1000research.52885.2)
Supplement: Peer review history

# Peer review: Reviewer response for version 2

**DOI:** 10.5256/f1000research.77259.r94734

**Published:** 2021-09-20

**Authors:** Federico Longhini

**Affiliations:** 1 Anesthesia and Intensive Care, Department of Medical and Surgical Sciences, Magna Græcia University, Catanzaro, Italy

No further comments on my side.

Are the rationale for, and objectives of, the Systematic Review clearly stated?

Yes

Is the statistical analysis and its interpretation appropriate?

Yes

Are sufficient details of the methods and analysis provided to allow replication by others?

Yes

Are the conclusions drawn adequately supported by the results presented in the review?

Yes

Reviewer Expertise:

NA

I confirm that I have read this submission and believe that I have an appropriate level of expertise to confirm that it is of an acceptable scientific standard.

# Peer review: Reviewer response for version 1

**DOI:** 10.5256/f1000research.56213.r89642

**Published:** 2021-07-29

**Authors:** Federico Longhini

**Affiliations:** 1 Anesthesia and Intensive Care, Department of Medical and Surgical Sciences, Magna Græcia University, Catanzaro, Italy

I have reviewed the manuscript and there are some criticism requiring discussion:

1. Please note that at least two articles should be included in the review for HFNC vs NIV for the primary outcome (Sklar 2018 and Papachatzakis 2020).
2. I suggest to check for other articles in the review by Pisani et al (PMID: 31591056), that also merits to be cited in the manuscript.
3. Please update findings and discussion according to the aforementioned points.
4. "While this could suggest that HFNT is non-inferior to NIV, HFNT cannot be recommended as an alternative management strategy to reduce PaCO _2_ due to the low quality of evidence, lack of standardization of time-points for PaCO _2_ measurement and the lack of adequately powered sample sizes." I would mitigate this message. In case of failure of NIV due to interface intolerance, with improving blood gases, I would  attempt to shift the treatment to HFNC, in order to avoid intubation. noteworthy, several studies have demonstrated that delay in intubation for hypercapnic respiratory failure does not impact on patients' outcome and survival.

Are the rationale for, and objectives of, the Systematic Review clearly stated?

Yes

Is the statistical analysis and its interpretation appropriate?

Yes

Are sufficient details of the methods and analysis provided to allow replication by others?

Yes

Are the conclusions drawn adequately supported by the results presented in the review?

Yes

Reviewer Expertise:

Respiratory failure, HFNC, NIV, invasive mechanical ventilation

I confirm that I have read this submission and believe that I have an appropriate level of expertise to confirm that it is of an acceptable scientific standard, however I have significant reservations, as outlined above.

## References

[1] : The Impact of High-Flow Nasal Oxygen in the Immunocompromised Critically Ill: A Systematic Review and Meta-Analysis.*Respir Care*.2018;63(12) : 10.4187/respcare.059621555-156610.4187/respcare.0596230467226

[2] : High-Flow Oxygen through Nasal Cannula vs. Non-Invasive Ventilation in Hypercapnic Respiratory Failure: A Randomized Clinical Trial. *International Journal of Environmental Research and Public Health*.2020;17(16) : 10.3390/ijerph1716599410.3390/ijerph17165994PMC745968732824771

[3] : High flow through nasal cannula in exacerbated COPD patients: a systematic review.*Pulmonology*.25(6) : 10.1016/j.pulmoe.2019.08.001348-35410.1016/j.pulmoe.2019.08.00131591056

# Response

**Published:** 2021-09-01

**Authors:** Asem Alnajada

**Affiliations:** Queen's University Belfast, UK

We would like to thank the reviewer for his time in reviewing this manuscript. The comments have been responded to individually as stated below. We feel that the manuscript has improved with his input and hope that he is satisfied by our response and additional changes.

**C1:** Please note that at least two articles should be included in the review for HFNC vs NIV for the primary outcome (Sklar 2018 and Papachatzakis 2020).

**R1:** Thank you for this comment, the studies cited have their merits in exploring the role of HFNT for AHRF. However, the studies cited don’t fit our inclusion criteria which have been described in the protocol published in the PROSPERO database ( CRD42019148748 ) and the methodology section of the systematic review, therefore, cannot be included in our SR. Specifically, the systematic review focusses on studies that have utilised HFNO as the initial management strategy for acute hypercapnic respiratory failure (AHRF). This systematic review is unique in that respect as evidence synthesis in this emergency clinical scenario is lacking with studies that have utilised at later stages of management such as post initial NIV use, interspersed with NIV and indeed studies not limited to AHRF are included in previous systematic reviews.

 Sklar *et al* have conducted a systematic review to investigate the impact of HFNT for patients with immunocompromise which don’t meet most of the inclusion criteria which we established as our review include only randomized controlled trials, uncontrolled trials and cohort studies focusing on HFNT as initial treatment when compared to LFO and/or NIV for AHRF. Papachatzakis et al was excluded due to various reasons including the inclusion of a mixed population and did not utilise HFNT as an initial treatment plan for the patient.

**C2:** I suggest to check for other articles in the review by Pisani et al (PMID: 31591056), that also merits to be cited in the manuscript.

**R2:** Thank you for the comment. We have cited the review by Pisani *et al* whose group have done a lot of work in this area. The various papers included in that review were also captured through our search and included in our review if they conformed to our protocol published in the PROSPERO database.

**C3:** Please update findings and discussion according to the aforementioned points.

**R3:** The citations suggested are outside the scope of the systematic review inclusion criteria and hence not included for outcome analysis. To give a broader picture of the field and the scope of HFNO, we have amended the background section to include the articles suggested above.

**C4:** While this could suggest that HFNT is non-inferior to NIV, HFNT cannot be recommended as an alternative management strategy to reduce PaCO2 due to the low quality of evidence, lack of standardization of time-points for PaCO2 measurement and the lack of adequately powered sample sizes." I would mitigate this message. In case of failure of NIV due to interface intolerance, with improving blood gases, I would  attempt to shift the treatment to HFNC, in order to avoid intubation. noteworthy, several studies have demonstrated that delay in intubation for hypercapnic respiratory failure does not impact on patients' outcome and survival.

**R4:** Thank you for this comment. The current evidence base to suggest HFNO in patients’ failing NIV is limited to small studies with no definitive efficacy studies. The authors do agree that it is an area that requires immediate attention. The paragraph has been amended to reflect the reviewers’ comments. “While this could suggest that HFNT is non-inferior to NIV, HFNT cannot be recommended as an alternative management strategy to reduce PaCO2 due to the low quality of evidence, lack of standardization of time-points for PaCO2 measurement and the lack of adequately powered sample sizes. Similarly, in patients failing NIV due to compliance issues, HFNO may be a promising option to limit mechanical ventilation. This recommendation falls beyond the scope of this systematic review and is a clinical scenario that requires urgent attention".

 We once again thank the reviewer for the time and effort taken in reviewing the manuscript and providing the comments.

# Peer review: Reviewer response for version 1

**DOI:** 10.5256/f1000research.56213.r87742

**Published:** 2021-07-14

**Authors:** Ben Messer

**Affiliations:** 1 North East Assisted Ventilation Service, Newcastle Upon Tyne Hospitals NHS Foundation Trust, Newcastle upon Tyne, UK

This is a thorough systematic review which has appropriate methodology and identifies the paucity of evidence available comparing HFNO to NIV.  There are minimal differences detectable and no clinically important differences.

 The conclusions drawn are appropriate and importantly, mention is made of the benefits of NIV which provide a rationale for further study including a trial of NIV vs HFNO.

Are the rationale for, and objectives of, the Systematic Review clearly stated?

Yes

Is the statistical analysis and its interpretation appropriate?

Yes

Are sufficient details of the methods and analysis provided to allow replication by others?

Yes

Are the conclusions drawn adequately supported by the results presented in the review?

Yes

Reviewer Expertise:

Acute respiratory support

I confirm that I have read this submission and believe that I have an appropriate level of expertise to confirm that it is of an acceptable scientific standard.

# Response

**Published:** 2021-09-01

**Authors:** Asem Alnajada

**Affiliations:** Queen's University Belfast, UK

We would like to thank the reviewer for his time in reviewing this manuscript. The comments have been responded to individually as stated below. We feel that the manuscript has improved with his input and hope that he is satisfied by our response and additional changes.

**C1:** This is a thorough systematic review which has appropriate methodology and identifies the paucity of evidence available comparing HFNO to NIV. There are minimal differences detectable and no clinically important differences.

**R1:** Thank you for this comment, as you mentioned the paucity has been identified in HFNO vs NIV. This is an important point to mention as this is currently a signal that HFNO is non-inferior to NIV in managing mild to moderate acute type 2 respiratory failure but the evidence base is poor and important clinical outcomes need to be robustly investigated.

**C2:** The conclusions drawn are appropriate and importantly, mention is made of the benefits of NIV which provide a rationale for further study including a trial of NIV vs HFNO.

**R2:** We agree with the rational comment you had given. Further studies are required to thoroughly evaluate the clinical significance between the treatment groups.

 We once again thank the reviewer for the time and effort taken in reviewing the manuscript and providing the comments.
